# The Report of the Committee on the Inebriates Acts—II

**Published:** 1909-01-23

**Authors:** 


### SPECIAL ARTICLE.

### THE REPORT OF THE COMMITTEE ON THE INEBRIATES ACTS—II.

The Report is presented under seven distinct headings. First is an Introduction on the Nature of Inebriety, in which the question is discussed whether inebriety can properly be considered to be a disease. The Committee regards this as " a question of nomenclature," but says there are cogent reasons why the term disease should not be used to characterise the inebriate habit; for it leads to the " erroneous and disastrous " inference that the habit " is to be accepted with fatalistic resignation, and that the inebriate is not to be encouraged to make any effort to amend his ways." After a brief History of Legislation concerning Inebriates, the Report considers the Habitual Drunkards Act, 1879, its Defects, and Proposed Amendments, and advocates, as both the preceding Committees—that of 1872 and that of 1892,—advocated, provision for the compulsory deprivation from intoxicants of those inebriates who do not commit offences and will not avail themselves of the voluntary provisions of the Act of 1879. The Report suggests that the omission of these compulsory measures from previous Acts is due in part to the unripeness of public opinion, and in part to the absence of definite safeguards against abuse. It considers that public opinion is now ripe for such measures, and suggests a definite mode of procedure, by which the inebriate shall first have the option of placing himself under a control which can be enforced if he refuses to submit voluntarily. The Report suggests, as the first step, a pledge, or voluntary obligation to abstain for a specified time of not less than a year from intoxicants, the difference between this statutory pledge, which is to be taken before a magistrate, and the pledge that can now be taken before anyone, being that if the statutory pledge is broken, the breach is to form a ground for the application of compulsory measures. The power to enter a Retreat voluntarily is preserved, and, in addition, it is recommended that a milder machinery, under the title of " Guardianship," should be provided. The Report suggests that an inebriate should be permitted to apply for the appointment of a guardian, who should have power to prescribe a residence for the inebriate, either in his own house or in that of the guardian; to prevent the inebriate from obtaining intoxicants; to subject him to the control of nurses; and to warn the sellers of drink and drugs against selling them to him. If the inebriate refuses to apply for the appointment of a guardian, or if the guardian finds his task impracticable, the judicial authority is to have power, on the application of a relative, friend, or voluntarily appointed guardian, to apply compulsion, in the form of a rather more stringent guardianship, or in that of committing the inebriate to a Retreat for a specified time. This is the third time that a Public Committee has recommended, in the strongest terms, the provision of compulsory powers for the control of inebriates who are not offenders against the law, and we trust that this time the recommendation will not be ignored. None but medical practitioners know the vast amount of misery that centres round the inebriate man or woman, who makes the home a hell, but yet never commits a public offence or comes into the hands of the police. It is a scandal that such persons cannot be dealt with except with their own consent, which is practically never given until the family is in a position to apply such moral pressure as virtually amounts to compulsion; and we trust that, whatever other recommendations of the Committee are ignored, these at least will be embodied in an Act of Parliament.

The report recommends further a drastic amendment of the Inebriates Act, 1898, which deals with inebriates who commit offences. Out of 1,751,820 persons convicted of drunkenness since the Act has been in force only 2,600 have been dealt with under the Act. For this failure the Committee finds six efficient causes. Some magistrates overlook the Act, others are unwilling to put in in force; the definition of "habitual drunkard" has been so interpreted that very few habitual drunkards can be brought within it; the necessity of proving three previous convictions for drunkenness; the necessity of obtaining the consent of the inebriate to be dealt with summarily; lack of accommodation in reformatories; and the three years' sentence. All these obstacles would be removed if the recommendations of the Committee were adopted. They recommend a new definition of "inebriate," a term they prefer to habitual drunkard, and they frame the definition so as to include the slaves of drug habits, as well as of alcohol. They recommend the abolition of the necessity of proving three previous convictions, which they regard as unnecessary and misleading. They would abolish the option of the drunkard to go to assizes or sessions, and they advise a scheme of graduated sentences, mitigated in every case by release on probation. But the most drastic recommendation is that to remedy the lack of accommodation. There is room in existing reformatories for only a small fraction of the inebriates who are fit to be committed by the courts, and, as long as this lack prevails, the Act cannot be efficiently enforced. Reformatories are at present provided by local authorities, by philanthropic societies, by private persons, and by the State. The Committee proposes that in future all reformatories should be provided by the State, and all inebriates in them supported by the State; that the whole service should be an obligation on the State, and should be administered as the prison service now is. The subject of finance is considered at length in the report, and reason is shown for the belief that administration by the State would be more economical than that by local authorities, whose expenditure is declared to be in some cases unjustifiable, and a waste of public money. The report is interesting, carefully reasoned and most exhaustive.

